# The prevalence and associated factors of symptomatic cervical Spondylosis in Chinese adults: a community-based cross-sectional study

**DOI:** 10.1186/s12891-018-2234-0

**Published:** 2018-09-11

**Authors:** Yanwei Lv, Wei Tian, Dafang Chen, Yajun Liu, Lifang Wang, Fangfang Duan

**Affiliations:** 1 Department of Epidemiology and Biostatistics, Public Health College, Peking University, 38# Xueyuan Road, Haidian district, Beijing, 100191 China; 2 Clinical Epidemiology Research Center, Beijing Jishuitan Hospital, 31# Xinjiekou Dongjie, West district, Beijing, 100035 China; 3 Department of Spine, Beijing Jishuitan Hospital, 31# Xinjiekou Dongjie, West district, Beijing, 100035 China

**Keywords:** Cervical spondylosis, Community-based, Cross-sectional study, Prevalence, Associated factors

## Abstract

**Background:**

Cervical spondylosis adversely affects life quality for its heavy disease burden. The report on the community-based prevalence and associated factors of cervical spondylosis is rare, especially in Chinese population. Whether prevention is needed and how to prevent it is not clear. This study aims to explore its prevalence and related lifestyle factors and provide evidence on prevention of cervical spondylosis.

**Methods:**

A community-based multistage cross-sectional survey of six communities from the Chinese population was conducted. A face-to-face interview was conducted to obtain individual information, and prevalence was calculated. Single-factor analysis and multivariable logistic regressions were used to explore the associated factors in total and subgroup populations.

**Results:**

A total of 3859 adults were analyzed. The prevalence of cervical spondylosis was 13.76%, although it differed significantly among the urban, suburban, and rural populations (13.07%, 15.97%, and 12.25%, respectively). Moreover, it was higher in females than in males (16.51% vs 10.49%). The prevalence among different age groups had an inverted U shape. The highest prevalence was in the age group from 45 to 60 years old. The associated factors differed by subgroups. There were positive associations between engaging in mental work, high housework intensity, and sleep duration of less than 7 h/day with cervical spondylosis. Going to work on foot was a negative factor of cervical spondylosis in the total population. For people aged less than 30 years, keeping the same work posture for 1–2.9 h/day was a special related factor. Exposure to vibration was an associated factor for females aged 45–60 years. Menopause was a special related factor for women.

**Conclusions:**

Prevalence of cervical spondylosis was high in Chinese population. People younger than 60 years were the focus of prevention for cervical spondylosis. Moreover, the characters between male and female and among different age groups were different and required targeted interventions.

## Background

Cervical spondylosis is a chronic degenerative process of the cervical spine. It affects the vertebral bodies and intervertebral disks of the neck and leads to herniated intervertebral disks, osteophytes, and ligament hypertrophy. This may eventually cause compression of the nerve roots and spinal cord [1]. Numbness, weakness, and tingling in the neck and/or arms, pain in the neck and/or arms, neck stiffness, and headaches are the usual symptoms of cervical spondylosis [2]. According to the reports, pain, numbness, and other symptoms were related to depression and insomnia [3, 4]. Although many asymptomatic adults have spondylotic changes according to the imaging examination, cervical spondylosis can cause stenosis of the spinal canal, lateral recess, and foramina and cause clinical symptoms, such as neck pain [5–7]. Neck pain was the most common symptom of cervical spondylosis [8]. The lifetime prevalence of the adult population was 48.5%, and the prevalence of screen-using workers was 55% [9, 10]. According to the global burden of disease study of 2013 [11], in 301 acute and chronic diseases and injuries in 188 countries, neck pain was one of the top 10 causes of years lived with disability. It ranked the fourth globally and the second in China, relatively. Cervical spondylosis not only affects the life quality but also increases the economic burden, since high-cost surgery is a regular treatment method. Therefore, cervical spondylosis might become a public health concern.

Cervical spondylosis is an age-related degeneration and chronic noncommunicable disease. Previous studies showed that age was the main risk factor and a contributor to the incidence of cervical spondylosis. The risk increased with aging [2, 12, 13]. Moreover, several other factors, such as occupational factors, exercises, and so on, exist [14]. In China, the prevalence and related factors were mainly hospital-based studies.However, the community-based prevalence and related factors of cervical spondylosis were rarely reported in Chinese population. With the social development of China and the onset of population aging, a large number of patients with cervical spondylosis may exist. The lifestyle has changed a lot in Chinese population,but the evidence of whether it needs to be controlled and how to control is lacking.

Therefore, the objective of this study was to report the baseline of the prevalence of cervical spondylosis and its related lifestyle influence factors of Chinese adults and provide evidence on its prevention and management.

## Methods

### Study participants

The present community-based cross-sectional study was conducted in December 2010. A multistage, stratified sampling method was used to select a representative sample of persons aged 18 years or older and living in Beijing for at least 6 months. The sampling process referred to a previous study [15]. The study protocol was approved by the institutional review board and the ethics committee of the Beijing Jishuitan Hospital, Beijing, China. Written informed consent was obtained from each participant before the data was collected.

### Diagnostic criteria

The patients of cervical spondylosis were self-reported doctor-diagnosed. Participants who had been diagnosed in the hospital were the patients. According to the consensus guide, cervical spondylosis was diagnosed by the clinical symptoms, signs and imaging examinations if needed in China. Clinical symptoms and signs were the diagnosis base of cervical spondylosis (Table 1) [16]. According to the clinician judgment, radiography or/and computed tomography or/and magnetic resonance imaging were used for the imaging examinations, and they were mandatory for diagnosing cervical spondylosis.

**Table 1:** The symptoms and signs of cervical spondylosis

symptoms	signs
Cervical pain aggravated by movement	Poorly localised tenderness
Referred pain (occiput, between the shoulder blades, upper limbs)	Limited range of movement (forward flexion, backward extension, lateral flexion, and rotation to both sides)
Retro-orbital or temporal pain (from C1 to C2)	Minor neurological changes like inverted supinator jerks (unless complicated by myelopathy or radiculopathy)
Cervical stiffness—reversible or irreversible	
Vague numbness, tingling, or weakness in upper limbs	
Dizziness or vertigo	
Poor balance	
Rarely, syncope, triggers migraine, pseudo-angina	

### Data collection

The face-to-face interview was used to collect the information. The information included sociodemographic information, such as place of residence, age, sex, per capita monthly income, education, type of medical insurance, and education level; physical measurement index, including body mass index (BMI, measured as weight in kgdivided by height in m^2^) and waist–hip ratio (WHR, the ratio of waist circumference and hip circumference); and lifestyle information containing smoking, drinking, nature of labor (physical-based, mental-based, or mixed), vibration, job posture, working intensity, duration of the same working posture during the day, transportation tools (nonmanpower transportation tool, bicycle, and walking), housework intensity, exercise frequency, exercise intensity, and sleep duration per day. People presently smoking at least one cigarette per day for at least 1 month or having used at least 100 cigarettes during lifetime were defined as smokers. The alcohol drinking group referred to persons whose alcohol consumption was 1000 mL of beer or 100 mL of liquor per week and lasting 1 year or more. Vibration denoted people operating a motor or a similar working environment in which movement was felt as a vibration. Body weight and height were measured by the researchers. A feasibility test of data collection and survey process optimization was done via a pilot study conducted prior to the actual study. A technical training of the investigators was conducted before being sent to interview the participants. Data were doubly entered in parallel using the EpiData 3.1 software (The EpiData Association, Odense, Denmark).

### Statistical analysis

Prevalence was reported with a standard error. Area-, age-, and sex-specific prevalences were also reported. Moreover, the prevalence associated with different education levels, BMI, nature of labor, income, drinking, smoking, job posture, transportation tools, sleep duration, vibration, duration of working posture, exercise frequency, exercise intensity, WHR, and menopause of women subgroup was calculated. The single-factor analysis was examined by *χ*^2^ tests or the Kolmogorov–Smirnov test. The multivariate analysis of associated factors was analyzed by the multivariable logistic regression model in the total population and in gender and age subgroup populations. Only significant variables in the single factor analysis were included in the multivariable model. The variables were selected by stepwise method. All *p* values were two tailed, not adjusted for multiple tests, and considered significant at *p* < 0.05. All statistical tests were carried out using the SPSS 18.0 software (SPSS Inc., IL, USA).

## Results

### Participant demographics

The study included 3900 participants, of which 3888 completed the study and 3859 had adequate disease information and were included in the analysis finally. The response rate was 99.7% in total, and 99.6% for males and 99.8% for females. Among the participants, 1820 were males (47.27%) and 2029 were females (52.73%). The mean age was 45.85 ± 16.19 years.

### Prevalence of cervical Spondylosis

**Fig. 1:**
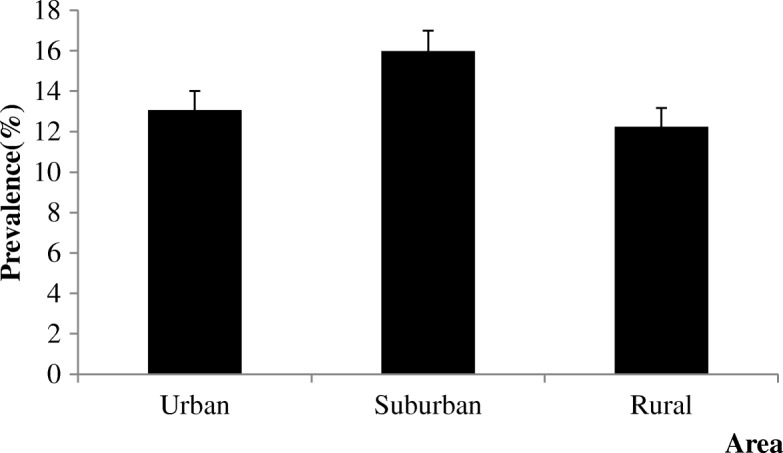
The area specific prevalence of cervical spondylosis (I bar indicates prevalence ± standard error)

**Fig. 2:**
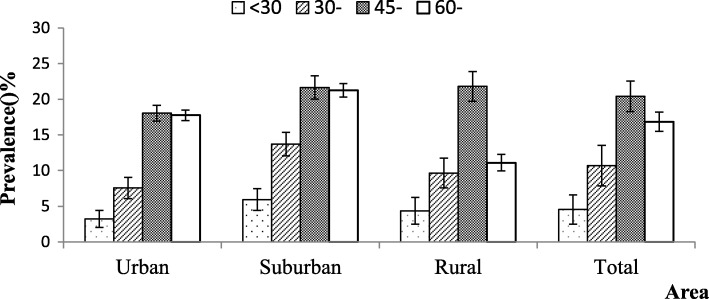
The age-specific prevalence of cervical spondylosis in different areas (I bar indicates prevalence ± standard error)

**Fig. 3:**
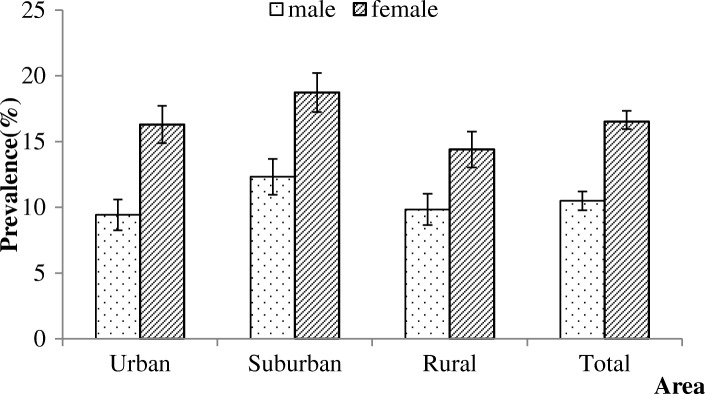
The sex-specific prevalence of cervical spondylosis in different areas (I bar indicates prevalence ± standard error)

Among 3859 subjects, 531 were diagnosed with cervical spondylosis, and the prevalence of cervical spondylosis was 13.76% [95% confidence interval (CI): 12.67–14.85%)]. The prevalence of cervical spondylosis in the suburban (15.97%) area was higher than that in the urban and rural areas (13.07% and 12.25%; *p =* 0.016; Fig. 1). The prevalence presented a rising trend with increasing age (*P* < 0.001; Fig. 2). The females had a higher prevalence of cervical spondylosis compared with the males (*P* < 0.001; Fig. 3). Participants with less education had a higher prevalence of cervical spondylosis (*P* < 0.001). The prevalence of cervical spondylosis increased with increasing BMI (*P =* 0.001). A significant difference in the prevalence was found among the three kinds of transportation modes (*P =* 0.013). The sleep duration less than 7 h/day had a higher prevalence compared with those sleeping for no less than 7 h/day (*P* < 0.001). People holding the same working posture for about 1–2.9 h were more likely to experience cervical spondylosis (*P =* 0.015). The prevalences of higher and lower exercise frequencies groups were both higher than other group (*P* < 0.001). Exercise intensity was related to the prevalence of cervical spondylosis (*P* < 0.001). People whose WHR was central obesity had a higher prevalence compared with the normal people (*P* = 0.002). Menopausal women had a higher prevalence than the nonmenopausal group (*P* < 0.001) (Table 2). Housework intensity was associated with cervical spondylosis (Table 3).

**Table 2:** Regional and Population-Based Distribution and Characteristics of Cervical spondylosis

	N	n	p	Sp	χ^2^	p
Place of residence						
Urban	1293	169	13.07	0.94	8.257	0.016
Suburban	1284	205	15.97	1.02		
Rural	1282	157	12.25	0.92		
Age(years)						
< 30	813	37	4.55	0.73	118.304	< 0.001
30-	1065	114	10.70	0.95		
45-	1199	245	20.43	1.16		
≥ 60	772	130	16.84	1.35		
Sex						
Male	1820	191	10.49	0.72	29.432	< 0.001
Female	2029	335	16.51	0.82		
Education						
Undergraduate or higher	598	60	10.03	1.23	18.503	< 0.001
Junior college	689	75	10.89	1.19		
Senior high school	1121	167	14.90	1.06		
Junior high school or lower	1414	225	15.91	0.97		
BMI**(**kg/m^2^**)**						
< 18.5	235	15	6.38	1.60	15.559	0.001
18.5-	1912	250	13.08	0.77		
24.0-	1324	201	15.18	0.99		
≥ 28.0	366	59	16.12	2.92		
Nature of labor						
Physical-based	1466	202	13.78	0.90	2.785	0.426
Mental-based	919	140	15.23	1.19		
Mixed	894	114	12.75	1.12		
Per capita monthly income level (¥)						
< 2000	2194	300	13.67	0.73	0.765	0.682
2000-	1400	198	14.14	0.93		
5000-	217	26	12.98	2.21		
Drinking						
Yes	768	115	14.97	1.29	1.191	0.275
No	3091	416	13.46	0.61		
Smoking						
Yes	929	110	11.84	1.06	3.798	0.051
No	2930	421	14.37	0.65		
Job posture						
Sitting	1280	180	14.06	0.97	5.746	0.219
Standing	799	104	13.02	1.19		
Frequently stooping	216	37	17.13	2.57		
Moving	1156	166	14.36	1.03		
Other	405	44	10.86	1.55		
Daily transportation tools						
Non manpower transportation tool	2116	271	12.81	0.73	8.744	0.013
Bicycle	743	127	17.09	1.38		
On foot	992	132	13.31	1.08		
Sleep duration per day (hours/day)						
≥ 7	3002	374	12.46	0.60	28.354	< 0.001
< 7	762	152	19.95	1.45		
Vibration						
Yes	334	50	14.97	2.96	0.451	0.502
No	3525	481	13.65	0.58		
Duration of the same work posture (hr/d)						
< 1	981	124	12.64	1.06	10.440	0.015
1-	987	164	16.62	1.19		
2-	477	69	14.47	1.61		
≥ 3	1411	174	12.33	0.88		
Exercise frequency						
≥ 2 times/week	1107	201	18.16	1.16	35.174	< 0.001
1 time/week	281	41	14.59	2.11		
1 time/2 weeks	77	8	10.39	3.50		
≤ 1 time/month	37	10	27.03	7.40		
No exercise	2336	267	11.43	0.66		
Exercise intensity						
None	2389	272	11.39	0.65	45.389	< 0.001
Mild	759	150	19.76	1.45		
Moderate	158	36	22.78	3.35		
Vigorous	553	73	13.20	1.44		
Waist–hip ratio						
Normal	1602	187	11.67	0.80	9.369	0.002
Central obesity	2243	339	15.11	0.76		
Menopause						
Yes	775	188	24.26	1.54	52.193	< 0.001
No	1145	134	11.70	0.95

**Table 3:** The Influence of Working Intensity and Housework Intensity on Cervical spondylosis

	Cervical spondylosis		Not Cervical spondylosis		Kolmogorov-Smirnov Z	*P*
	meadian	QL	meadian	QL		
Working intensity	5	3	5	3	1.145	0.145
Housework intensity	3	3	3	3	2.918	< 0.001

### Associated factors for cervical Spondylosis in the Total population

People living in the suburban area, those 30 years or older, females, those engaged in mental work, housework intensity, and those sleeping for less than 7 h/day had a positive association with cervical spondylosis. Going to work on foot was a negative associated factor for cervical spondylosis; the odds ratio (OR) was 0.690 (95% CI: 0.512–0.929) (Table 4).

**Table 4:** Associated Factors of Cervical spondylosis by Multivariable Logistic Regression

	β	S.E	Wald	P	OR	95% CI	
						lower	upper
Place of residence			7.172	0.028			
Urban					1.000		
Suburban	0.383	0.147	6.809	0.009	1.467	1.100	2.957
Rural	0.302	0.160	3.544	0.060	1.352	0.988	1.851
Age(years)			68.947	< 0.001			
< 30					1.000		
30-	0.845	0.226	13.980	< 0.001	2.327	1.495	3.623
45-	1.668	0.224	55.351	< 0.001	5.303	3.417	8.229
≥ 60	1.552	0.241	41.538	< 0.001	4.722	2.945	7.571
Sex							
Male					1.000		
Female	0.591	0.120	24.146	< 0.001	1.805	1.426	2.285
Nature of labor			10.446	0.005			
Physical-based					1.000		
Mixed	0.207	0.157	1.742	0.187	1.230	0.905	1.671
Mental-based	0.502	0.157	10.260	0.001	1.653	1.215	2.247
Housework intensity	0.086	0.026	10.850	0.001	1.090	1.035	1.147
Modes of daily transportation			6.014	0.049			
Non manpower transportation tool					1.000		
Bicycle	−0.089	0.151	0.348	0.555	0.915	0.681	1.229
On foot	−0.371	0.152	5.966	0.015	0.690	0.512	0.929
Sleep duration per day (<7 h/day)	0.383	0.132	8.410	0.004	1.466	1.132	1.899

### Associated Factors for Cervical Spondylosis in the Gender Subgroup

The associated factors for cervical spondylosis were significantly different between men and women. For males, age 30 years or older and having vibration characteristics during their working environment were associated factors for cervical spondylosis. For females, place of residence, age, menopause, mental-based work, housework intensity, and sleeping for less than 7 h/day were associated factors for cervical spondylosis, whereas going to work on foot was a protective associated factor for cervical spondylosis (Table 5).

**Table 5:** Epidemiological Characteristics of Cervical spondylosis in Male and Female Populations

	β	Se	Wald	P	OR	95% CI	
						lower	upper
Males							
Age(years)			25.211	< 0.001			
< 30					1.000		
30-	1.027	0.383	7.209	0.007	2.793	1.320	5.911
45-	1.496	0.369	16.401	< 0.001	4.465	2.164	9.210
≥ 60	1.733	0.380	20.749	< 0.001	5.657	2.684	12.923
BMI(kg/m^2^)			4.918	0.178			
18.5-					1.000		
< 18.5	−1.676	1.021	2.693	0.101	0.187	0.025	1.385
24-	0.152	0.189	0.649	0.421	1.164	0.804	1.687
≥ 28	0.388	0.299	1.679	0.195	1.474	0.820	2.649
Vibration(yes)	0.471	0.218	4.689	0.030	1.603	1.046	2.450
Females							
Place of residence			4.958	0.084			
Urban					1.000		
Suburban	0.442	0.211	4.389	0.036	1.556	1.029	2.354
Rural	0.413	0.235	3.097	0.078	1.512	0.954	2.397
Age(years)			46.433	< 0.001			
< 30					1.000		
30-	0.654	0.279	5.505	0.019	2.924	1.114	3.323
45-	1.644	0.273	36.222	< 0.001	5.177	3.031	8.843
≥ 60	1.271	0.298	18.135	< 0.001	3.565	2.986	6.399
Menopause(Yes)	0.572	0.217	6.983	0.008	1.772	1.159	2.710
Nature of labor			5.983	0.050			
Physical-based					1.000		
Mixed	0.411	0.225	3.339	0.068	1.508	0.971	2.344
Mental-based	0.522	0.222	5.537	0.019	1.686	1.091	2.605
Housework intensity	0.088	0.038	5.291	0.021	1.092	1.013	1.176
Daily transportation tools			10.013	0.007			
Non manpower transportation tool					1.000		
Bicycle	−0.169	0.211	0.638	0.424	0.845	0.558	1.278
On foot	−0.650	0.207	9.916	0.002	0.522	0.348	0.782
Sleep duration per day (<7 h/day)	0.474	0.187	6.401	0.011	1.606	1.113	2.318

### Associated factors for cervical Spondylosis in the age subgroup

The characteristics of cervical spondylosis differed by age groups. For the people aged less than 30 years, work intensity, keeping the same work posture 1–2.9 h/day and the gender were the associated factors for cervical spondylosis. For the group between 30 years and 45 years old, housework intensity was the only associated factor. For 45 and 60 years group, living in the rural area, female gender, engaging in mental work, sleeping less than 7 h/day and vibration exposure in working condition were the associated factors. Going to work on foot was a protective factor for cervical spondylosis. For people aged no less than 60 years, housework intensity, engaging in mental work and mixed work, and daily sleep duration less than 7 h were the associated factors for cervical spondylosis (Table 6).

**Table 6:** Epidemiological Characteristics of Cervical spondylosis among Different Age Groups

	β	Se	Wald	P	OR	95% CI	
						lower	upper
< 30 years group							
Work intensity	0.329	0.091	13.242	< 0.001	1.390	1.164	1.660
Duration of the same work posture (hours)			9.144	0.027			
< 1					1.000		
1-	2.527	1.049	5.805	0.016	12.522	1.602	97.850
2-	2.169	1.078	4.050	0.044	8.750	1.058	72.346
≥ 3 h	1.629	1.038	2.462	0.117	5.099	0.666	39.013
Gender(Female)	0.917	0.427	4.614	0.032	2.501	1.084	5.771
30-years group							
Housework intensity	0.111	0.052	4.530	0.033	1.117	1.009	1.237
45- years group							
Place of residence			8.023	0.018			
Urban					1.000		
Suburban	0.387	0.239	2.619	0.106	1.473	0.921	2.355
Rural	0.696	0.246	8.020	0.005	2.006	1.239	3.248
Gender(Female)	1.085	0.193	31.782	< 0.001	2.961	2.030	4.318
Nature of labor			7.135	0.028			
Physical-based					1.000		
Mixed	0.067	0.248	0.072	0.788	1.069	0.657	1.739
Mental-based	0.616	0.243	6.439	0.011	1.852	1.151	2.980
Daily transportation tools			6.017	0.049			
Non manpower transportation tool					1.000		
Bicycle	−0.109	0.217	0.252	0.615	0.897	0.586	1.372
On foot	−0.561	0.233	5.793	0.016	0.571	0.361	0.901
Sleep duration per day (<7 h/day)	0.506	0.194	6.771	0.009	1.658	1.133	2.426
Vibration(Yes)	−0.563	0.276	4.156	0.041	0.570	0.332	0.979
≥60 years group							
Nature of labor			7.872	0.020			
Physical-based					1.000		
Mental-based	0.733	0.294	6.237	0.013	2.082	1.171	3.703
Mixed	0.667	0.294	5.153	0.023	2.948	1.095	3.464
Housework intensity	0.136	0.054	6.426	0.011	1.145	1.031	1.272
Sleep duration per day (<7 h/day)	0.518	0.254	4.166	0.041	1.679	1.021	2.760

## Discussion

This was an epidemiological study of cervical spondylosis in Chinese population. The prevalence of cervical spondylosis was 13.76%, which was higher than the prevalence of diabetes (9.7%) [17]. According to the data of the population census in 2011, approximately 2.75 million patients suffered from cervical spondylosis in Beijing, a city having a population of 20 million. The prevalence of cervical spondylosis was high not only in the Chinese population but also in other areas of the world. According to a cohort study, the incidence of cervical spondylosis was 13.1% overall, in a total of 47,560 patients [18]. A study in the southwest region of Nigeria found a prevalence of 10.7% for cervical spondylosis [19], which was similar to the results of the present study. This indicated that cervical spondylosis was a major public health problem that needed a large-scale intervention.

The prevalence among different age groups had an inverted U shape. The group aged between 45 years and 60 years had the highest prevalence. This might cause absence from work because of the symptoms caused by cervical spondylosis, such as neck pain and so on [20–22]. Therefore, this group needed more preventive measures. Several factors might affect cervical spondylosis. Irrespective of the case–control or longitudinal study, age was an important related factor for cervical spondylosis [23, 24]. These results were consistent with the findings of the present study. Moreover, the present study also revealed that the strength of association in different age groups was different. Compared with the youngest age group, the adjusted OR was the highest in people aged between 45 and 60 years (OR = 5.303, 95% CI: 3.417–8.229) and second highest in those aged more than 60 years (OR = 4.722, 95% CI: 2.945–7.571). Several reasons might explain this. The first and the most important reason is that the characters might be different in the four age groups (Table 6). For example, for the youngest people aged less than 30 years, work intensity and keeping the same working posture for 1–3 h/day were the associated factors for cervical spondylosis. For people aged 30–45 years, housework intensity was the only associated factor for cervical spondylosis. This result indicated that the prevention measures should be different for different age groups. Second, the occurrence of cervical spondylosis as a chronic disease was the result of the long-term effect of the aforementioned factors. Considering the hysteresis effect, people younger than 60 years were the focus of prevention.

According to the report of Singh S et al., sex showed no significance with cervical spondylosis [23]. Sex was related with cervical spondylosis in this study. In the analysis of gender subgroups, age 30 years or older and vibration exposure in work environment were independent associated factors for cervical spondylosis in males; age 30 years or older, exposure to vibration during their daily work, menopause, those engaged in mental work, housework intensity, and those sleeping for less than 7 h/day were also the associated factors for cervical spondylosis for females, and going to work on foot was a protective factor for cervical spondylosis. The associated factors for females might be related to physiological characteristics and their division of labor.

Work-related factors, such as carrying head loads, were associated with cervical spondylosis [25–27]. For the youngest people aged less than 30 years, work intensity and keeping the same work posture ranging from 1 to 3 h/day were the associated factors for cervical spondylosis in the present study. Work intensity and the duration of keeping the same work posture were both indicators of neck loading. For this group of people, the cervical spine activity during the appropriate time interval might have some effects, such as turning the head. Occupation, sedentary lifestyle, and unhealthy working posture were the risk factors for cervical spondylosis [28]. For people younger than 30 years, those holding the same work posture for 1–1.9 h and 2–2.9 h had positive relation with cervical spondylosis, respectively, compared with those who held the same posture for less than 1 h. The present study did not find a significant difference between people holding the same work posture for more than 3 h and those holding the same posture less than 1 h. It might be due to the adaptability of the human body. The present study suggested that the appropriate activity time interval was 1 or less than 1 h.

Occupational low back pain is strongly associated with vibration [29–33]. A positive dose relationship exists between them [34]. The whole-body vibration during daily work was an associated factor of cervical spondylosis in males in this study. Although no report demonstrated the relationship between vibration and cervical spondylosis, occupational factors contributing to the acceleration of spinal degeneration included vehicle driving [35]. Vehicle driving caused vibration. Vibration could cause bone metabolism disorder and bone damage of lumbar vertebra [36]. Therefore, the whole-body vibration was related with the cervical spine. This result could explain why people who traveled by foot had lower prevalence than those who used nonmanpower transportation tool to some degree. Also, going to work on foot gave the body some exercise. Although some exercises had higher risk, some had no risk and others have protective effect [22, 37–41], on foot had positive association on cervical spondylosis in this study.

One study found menopause as an associatedfactor for cervical spondylosis (OR = 1.772, 95% CI: 1.159–2.710). Estrogens can maintain collagens that protect the intervertebral disk [42]. Lou et al. found a relationship between menopause and the degeneration of intervertebral disk [43]. Moreover, the changes in hormone levels during menopause could lead to the degeneration of vertebral endplate, which affected the nutritional distribution of the intervertebral disk, ending in the degeneration of the spine [44]. Females in the perimenopausal period need some health interventions to protect their spine health.

BMI ≥28 kg/m^2^ had a higher risk for lumbar osteoarthritis in the present study [15]. Obesity was a risk factor for intervertebral disk degeneration and spine disease [45, 46]. Obesity increased the weight of the skeleton and accelerated the intervertebral disk degeneration [47]. Besides, obesity was an inflammatory disorder that could cause the degeneration of intervertebral disk [48]. However, according to a 10-year cohort study [22], no association was found between obesity and degenerative cervical disease. In this study, the prevalence of cervical spondylosis was significantly different in different BMI groups and between normal and central obese groups according to the single-factor analysis. However, obesity and central obesity had no relation to cervical spondylosis in the multivariate analysis. This was probably because the anatomical position of the cervical vertebra was high in the body. It was less influenced by the weight of the body.

Moreover, the negative association between sleeping duration and cervical spondylosis was significant. This was possibly due to biomechanics and emotions. Weight loading was one of the important causes of spinal degeneration [49]. Short hours of sleep per day increased the weight loading time of the spine, thus accelerating its degeneration. Shorter sleep duration per day was associated with emotional stress. Emotional stress was associated with neck pain [50]. Neck pain usually was one of the possible symptoms in the process of cervical spondylosis.

The present study had two limitations. First, the prevalence of cervical spondylosis might have been underestimated because of the definition of cervical spondylosis. In this study, patients with cervical spondylosis were people not only having imaging changes but also having clinical symptoms. Therefore, people even having cervical degeneration imaging changes but no clinical symptoms were excluded. However, according to the current clinical guideline, people having not only imaging changes but also clinical symptoms needed treatment. In addition, if people don’t diagnosed in hospital, they were not classified to cervical spondylosis patients.There may be more cervical spondylosis patients in Chinese population. Therefore, the influence of underestimation on policy decisions was less. Second, regarding the evidence level and the relevant strength of the cross-sectional study, a cohort study is needed to provide further evidence of the correlation between the associated factors and cervical spondylosis. However, our findings supplemented the information of cervical spondylosis prevalence in community people which has received little attention from other studies. At the same time, we also reported the prevalence and associated factors in both middle-young and old age group, which was scarcely reported before. Since the middle-young-aged population were the main social labor and should be the focal point for the prevention of cervical spondylosis. Our findings would provide valuable information for the prevention of the disease on lifestyle,especially for middle-young-aged population.

## Conclusions

The prevalence of cervical spondylosis was high in Chinese population. People younger than 60 years were the focus of prevention. Moreover, the characters of between male and female and among different age groups were different and required targeted interventions.
